# Sustainability Perspectives of *Vigna unguiculata* L. Walp. Cultivation under No Tillage and Water Stress Conditions

**DOI:** 10.3390/plants9010048

**Published:** 2019-12-30

**Authors:** Lorenzo Guzzetti, Andrea Fiorini, Davide Panzeri, Nicola Tommasi, Fabrizio Grassi, Eren Taskin, Chiara Misci, Edoardo Puglisi, Vincenzo Tabaglio, Andrea Galimberti, Massimo Labra

**Affiliations:** 1 Department of Biotechnology and Bioscience, University of Milan-Bicocca, Piazza della Scienza 2, 20126 Milano, Italy; lorenzo.guzzetti@unimib.it (L.G.); davide.panzeri@unimib.it (D.P.); nicola.tommasi@unimib.it (N.T.); andrea.galimberti@unimib.it (A.G.); massimo.labra@unimib.it (M.L.); 2 Department of Sustainable Crop Production, Università Cattolica del Sacro Cuore, Via Emilia Parmense 84, 29122 Piacenza, Italy; andrea.fiorini@unicatt.it (A.F.); vincenzo.tabaglio@unicatt.it (V.T.); 3 Department of Biology, University of Bari, Via Orabona 4, 70125 Bari, Italy; fabrizio.grassi@uniba.it; 4 Department for Sustainable Food Process, Università Cattolica del Sacro Cuore, Via Emilia Parmense 84, 29122 Piacenza, Italy; eren.taskin@unicatt.it (E.T.); chiara.misci1@unicatt.it (C.M.)

**Keywords:** conservation agriculture, no-tillage, climate change, drought stress

## Abstract

Nowadays, agriculture is facing the great challenge of climate change which puts the productivity of the crops in peril due to unpredictable rain patterns and water shortages, especially in the developing world. Besides productivity, nutritional values of the yields of these crops may also be affected, especially under low mechanization and the low water availability conditions of the developing world. Conservation agriculture (CA) is a topic of emerging interest due to the provision of adequate yields and reduced environmental impact, such as greenhouse gas emissions, by being based on three main principles: minimum soil disturbance (reduced or no tillage), cover crop maintenance, and crop rotation. The aim of this study was to assess the impact of CA management on the growth performance and the nutritional profile of cowpea (*Vigna unguiculata* L. Walp), a pulse of African origin, commonly known as black eye bean under field conditions. A field experiment was designed to assess the effect of conventional tillage (CT) and no-tillage (NT) combined with the usage of a set of cover crops, coupled to normal and deficient water regimes. Cowpea was revealed to be able to grow and yield comparably at each level of the treatment tested, with a better ability to face water exhaustion under CA management. After a faster initial growth phase in CT plots, the level of adaptability of this legume to NT was such that growth performances improved significantly with respect to CT plots. The flowering rate was higher and earlier in CT conditions, while in NT it was slower but longer-lasting. The leafy photosynthetic rate and the nutritional profile of beans were slightly influenced by tillage management: only total starch content was negatively affected in NT and watered plots while proteins and aminoacids did not show any significant variation. Furthermore, significantly higher carbon and nitrogen concentration occurred in NT soils especially at the topmost (0–5 cm) soil horizon. These findings confirm the capability of CA to enrich soil superficial horizons and highlight that cowpea is a suitable crop to be grown under sustainable CA management. This practice could be pivotal to preserve soils and to save agronomical costs without losing a panel of nutrients that are important to the human diet. Due to its great protein and aminoacidic composition, *V. unguiculata* is a good candidate for further cultivation in regions of the word facing deficiencies in the intake of such nutrients, such as the Mediterranean basins and Sub-Saharan countries.

## 1. Introduction

The Green Revolution (1960–1980s) was aimed at improving the agronomic productivity and nutritional features of the major staple crops worldwide (e.g., maize, rice, and wheat) [1]. Most of the crop varieties were selected to deal with emerging environmental and biotic stresses (i.e., desertification, nutrient-poor soils, and extreme temperatures) and were expected to produce yields several times higher than minor crops and the local varieties. Unfortunately, overcoming some of these obstacles was not always possible in a sustainable way and during the past three decades, cultivation practices have been demanding a higher and higher use of water and agrochemicals (e.g., fertilizers, pesticides, and herbicides), to enhance (or maintain) maximum crop yields [2,3]. Environmental hazards, the poor maintenance of long-term plant and soil productivity and the higher costs in terms of agrochemicals and energy consumption produced the modern crisis of agriculture. To address this crisis and environmental concerns of the consumers, in recent years, the principles of Sustainable Agriculture were continuously promoted worldwide [4,5]. Therefore, for the green revolution of the 21st century, the practices of Organic farming (OF) and Conservation Agriculture (CA) are deemed environmentally friendly approaches to agriculture. Traditionally, OF is based on the creation of the correct ecosystems for the crop productivity with a holistic approach that considers maintenance and health of the soil, plants, and livestock, with strictly regulated use of external inputs while focusing on farm production and recycling of needed products (e.g., composting wastes and green mulching) and the adoption of integrated strategies against plant pests. On the other hand, CA represents a set of three crop management principles: (i) direct planting of crops with minimum soil disturbance, (ii) permanent soil cover by crop residues and cover crops, (iii) crop rotation [6]. Through these strategies, CA guarantees an optimum environment for the rhizosphere to capture nutrients and water [5]. The adoption of no-till (NT) and the maintenance of a crop residue mulch on the surface have assumed an important role, especially in the geographical areas characterized by consistent rainfall and the consequent risk of soil leaching [7]. Although most of the production zones in the Mediterranean region are characterized by hot summers and rainy winters, global warming has been increasing the risk of (i) soil degradation due to soil losses in response to the greater drought and torrential rainfall; (ii) soil salinization due the increase of droughts, irrigation, and sea level; and (iii) soil carbon stock depletion because of the increase of temperature and drought [8]. Therefore, the application of CA principles has the potential in the Mediterranean regions to preserve soil structure and fertility, as well as to improve productivity and quality of crops [9,10,11].

Promotion and research on CA in many instances have focused on the first two principles, which are minimum soil disturbance/no-tillage and surface crop residue. Species belonging to Fabaceae could also enhance soil fertility thanks to the nitrogen-fixing symbionts. Moreover, there are several legume crops that are also able to grow under stress conditions [12], such as water/salt stress, and could be adapted to no-tilled soils. Sub-Saharan Africa is an important source of stress-resistant legume grain cultivars, such as species belonging to the genera *Vigna* and *Lablab* [13,14,15], and some of these could also be adapted to grow under the Mediterranean climatic conditions.

In this study, we selected *Vigna unguiculata* L. Walp (also known as cowpea) to investigate the ability of this species to grow in the Mediterranean region under CA conditions. The species was adequate for such study due to the fact that its beans are rich in proteins and carbohydrates and have relatively low-fat content [16]. Moreover, *Vigna unguiculata* beans show an aminoacidic pattern that is complementary to that of many foods consumed in the Mediterranean area, such as cereal grains. These aspects make *V. unguiculata* a ‘strategic’ crop for the Mediterranean diet. Furthermore, *V. unguiculata* is attracting the attention of consumers and researchers due to its beneficial health properties, including anti-diabetic, anti-cancer, anti-hyperlipidemic, anti-inflammatory, and anti-hypertensive properties [17].

Specifically, in this work, the response of *V. unguiculata* to NT soil management both with and without irrigation was investigated. Plant growth features and plant productivity, in terms of straw biomass and grain yield, were evaluated. At the same time, the response of the soil carbon (C) and nitrogen (N) stock to NT was verified. Emissions of greenhouse gases CO_2_ and N_2_O from NT soils are highly variable and depend on complex interactions among soil properties (i.e., soil water content, soil C and N), microbes, and the cultivated plant. Usually, the increased soil organic C (SOC) in surface layers of no-till soils is widely found but may not be associated with increased C sequestration throughout the soil profile [18]. Therefore, the evaluation of the relative carbon balance under NT vs. CT is essential to better estimate the potential of NT to sequester additional C into the soil. Furthermore, there is no accordance in the scientific literature about the effect of NT in N sequestration and dynamics [18].

Moreover, the metabolic features of the seeds, in terms of nutritional components after boiling (to imitate the conditions of consumption and the effective intake for humans), were assessed.

## 2. Material and Methods

### 2.1. Experimental Design and Treatments

A one-year field experiment was carried out on a long-term field study (initiated in 2010) at the CERZOO experimental research station in Piacenza (45°00′18.0″ N, 9°42′12.7″ E; 68 m above sea level), Po valley, Northern Italy. The soil is a fine, mixed, mesic, Udertic Haplustalf (Soil Survey Staff 2014), with a silty clay loam texture (sand 122, silt 462, and clay 416 g kg^−1^) in the upper layer (0–30 cm). The main physical-chemical properties of the soil are reported in Fiorini et al. [19]. The climate is temperate, and the mean annual temperature and precipitation are 12.2 °C and 890 mm, respectively. Climatic data were collected from an automated meteorological station positioned in the experimental field (Figure S1 in Supplementary Materials). The experimental design was a randomized complete block (RCB) with four repetitions and two tillage treatments: conventional tillage (CT) and no-tillage (NT). In detail, (i) CT included an autumn plowing (35 cm) and two passages of rotating harrow in spring (15–20 cm) to prepare the seedbed, and (ii) NT consisted of direct sowing on a soil untilled for 7 years using a double-disk opener planter for seed deposition. Between 2011 and 2017, the crop sequence was a three-year crop rotation, with soybean (*Glycine max* L. Merr.), durum winter wheat (*Triticum turgidum* L. var. durum), and maize (*Zea mays* L.). During winter off-seasons, a mixture of winter cover crops was sown in NT plots, right after harvesting the previous main crop. The species composing the cover crops mixture were rye (*Secale cereale* L.), hairy vetch (*Vicia villosa* L.), crimson clover (*Trifolium incarnatum* L.), Italian rye-grass (*Lolium multiflorum* Lam.), and radish (*Raphanus sativus* L.). In 2018, 15 m^2^ (5 m × 3 m) within each plot (1430 m^2^; 65 m × 22 m) were cropped with *V. unguiculata*, both under CT and NT. The experiment was established to compare responses of cowpea cultivation to contrasting tillage systems. NT and CT planters were calibrated in order to obtain the same sowing depth in both treatments. The distance between planting rows was 50 cm, and the distance between seeds on the same row was 10 cm. Sowing of cowpea was carried out on May 18th (sowing depth: 3–4 cm) and the harvest took place on August 9th. No fertilizers were applied during the growing season, and weeds were suppressed weekly by hands.

On July 20th, when cowpea plants were at the beginning of the blooming, each plot was divided into two subplots. The first one was sprinkler irrigated to prevent water stress (20 mm per time, for a total of three irrigation events), whereas, in the second sub-plot, any kind of natural or artificial water input was prevented by temporarily covering those sub-plots through greenhouses to induce and to simulate the dry season.

### 2.2. Biomass and Morphophysiological Sampling and Analysis

During the whole growing seasons, plants (N = 320) were measured weekly for a total of 7 surveys to detect the following parameters: the dimension of the canopy (cm), the total number of leaves, and number of flowers. Plants were labelled univocally in order to follow the growing performance of each individual over time. After the greenhouses settlement, to evaluate the role of water exhaustion on plants, in each subplot three plants were randomly chosen to be undergone to Pocket PEA Chlorophyll Fluorimeter (Hansatech Instruments, Pocket PEA, 2013) measurements, providing an estimate of the F_v_/F_m_ ratio. F_v_ is the fluorescence variable, calculated as F_m_ − F_o_, where F_o_ is the fluorescence origin and F_m_ is the fluorescence maximum.

Measurements took place from the 5th to the 7th survey on the same labelled plants in a time range between 10 a.m. and 1 p.m.

After the 7th survey, grain yield and above-ground biomass weight were measured by harvesting three randomly selected 2.0 × 1.0 m squares from each subplot. Above-ground biomass was manually cut at the soil level and weighed. Grain and straw were also separated. The dry weight biomass of cowpea (grain and straw) was gravimetrically determined by drying biomass at 70 °C until constant weight.

Fruits derived from the remaining plants were harvested and stored at −20 °C before phytochemical analyses.

### 2.3. Measurement of Soil Physical Properties

To determine soil C and N stock, soil bulk density (BD, 0–30 cm), SOC, and N concentration in the 0–20 cm layer (0–5 and 5–15 cm) were measured right after harvesting cowpea. Four randomly selected undisturbed soil core samples were collected on August 20th, 2018 from each subplot, using a steel auger of 5 cm diameter. Soil BD was determined according to the cylinder method [20], while samples to determine SOC and N concentration were air-dried, ground with a rubber pestle, and sieved to 2 mm. About 1 g of dry soil per each sample was weighed and used to determine C and N concentration by Dumas combustion method with an elemental analyzer varioMax C:N (VarioMax C:NS, Elementar, Germany). Soil carbonate removal was not necessary due to the low carbonate content in the soil.

**Figure 1:**
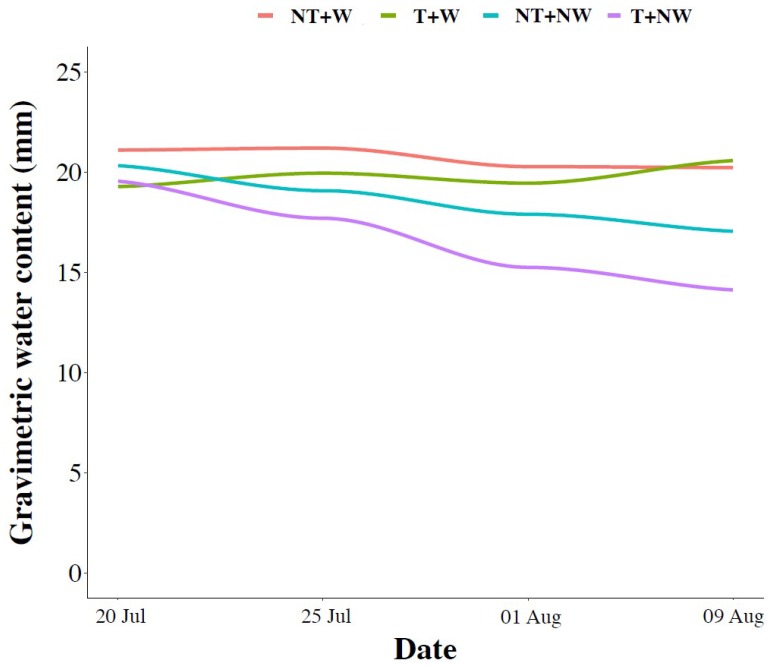
Trend of the gravimetric water content in the four different treatments. CT = conventional tillage, NT = no tillage, W = watered, NW = not watered.

To estimate the effect of watering on soil, the gravimetric water content was measured on a weekly basis, both in watered (W) a non-watered (NW) plots (Figure 1). From May 18th to July 20th precipitation events occurred for a total of 121 mm. After July 20th, in W sub-plots, precipitation and irrigation events consisted of 38 and 60 mm (three irrigations of 20 mm each), respectively.

### 2.4. Chemical Characterization of V. unguiculata Seeds

Phytochemical analysis was carried out on the cowpea seeds that were boiled in water for one hour and then were left to cool down for another hour, as suggested by Olaleke et al. [21], in order to mimic the conditions of cooking and consumption. Subsequently, seeds were dried at 50 °C overnight and then ground to a fine powder.

#### 2.4.1. Evaluation of Total Starch Content (TSC)

The TSC content was indirectly evaluated by measuring the amount of NADPH in samples after an enzymatic treatment by using the Kit Megazyme^®^ Total Starch AOAC Method 996.1 1 and AACC Method 76.13. Briefly, 50 mg of dry powder was added to 200 μL of ethanol 80% *v/v* and 1 mL of KOH 2 M and left stirring for 20 min at 4 °C. Then, 4 mL of a sodium acetate buffer 1.2 M Ph = 3.8 were added followed by the addition of 50 μL of α-amylase (8300 U/mL) and then 50 μL of amyloglucosidase (AMG, 3300 U/mL). Samples were incubated for half an hour at 50 °C and then centrifuged at 3000 rpm for 10 min to recover the supernatant. For each sample, the reaction mixture was prepared as follows in a quartz cell: 1 mL H_2_O, 25 μL of the supernatant, 50 μL of a buffer solution pH = 7.6, 50 μL NADP+/ATP. The solution was incubated for 3 min at room temperature and then the absorbance was read at 340 nm against the blank containing water instead of sample.

Then, 10 μL of a solution containing hexokinase (HK) and glucose-6-phosphate-dehydrogenase (G6PDH) was added. After an incubation of 5 min at room temperature, the absorbance was read against the blank again at 340 nm. TSC is expressed as g of total starch per 100 g of dry powder.

#### 2.4.2. Total Protein Content (TPC)

The extraction of amino acids and proteins from dry seeds was performed as in Olaleke et al. [21] with minor modifications. Briefly, 2 g of dry powder were extracted in 50 mL of an aqueous solution of theanine at a concentration of 10 μg/mL. Theanine was chosen as internal standard as it is not biosynthesized in beans. The solution was stirred at 500 rpm for 5 min. Then samples were centrifuged at 5000 rpm for 30 min and the supernatant was recovered and freeze-dried. Yields of extraction were recorded by weighing freeze-dried extracts.

The Total Protein Content (TPC) was evaluated by using the Bradford assay as follows: 1 mL of 50% Coomassie-Brilliant Blue Bradford reagent (ThermoFisher) was incubated at room temperature with 2 μL of extract of known concentration for a minute. Absorbance was read against blank at 595 nm and fitted on a calibration curve made up with BSA (Bovine Serum Albumin) in a range between 0 and 6 mg/mL. TPC was expressed as g total proteins per g of extract and was then multiplied per the yield of extraction to be expressed on g of dry powder.

#### 2.4.3. Amino Acidic Content and Characterization

The evaluation of the amino acid content was performed by High Performance Liquid Chromatography coupled to a Diode Array Detector (HPLC-DAD), 1260 Infinity II LC System (Agilent, 2018). A calibration curve was made up by using an amino acid mixed solution (Merck, analytical standard, 17 amino acids plus tryptophan) in a range between 0.078 mM and 1.25 mM. The column used for this analysis is an Agilent Poroshell HPH C18 (100 × 4.6 mm, 2.7 µm) with a guard column (AdvanceBio Oligo 4.6 × 5 mm, 2.7 µm) and it was kept at 40 °C. Mobile phases were phosphate buffer (10 mM Na_2_HPO_4_ pH = 8.2) and Acetonitrile:Methanol:Water (45:45:10). The elution program is (%B): 0–0.35 min 2%, 13.4 min 57%, 13.5 min 100%, 15.7 min 100%, 15.8 min 2%, 18 min end. Flow rate was constant at 1.5 mL/min. Solvents were HPLC grade, whereas the buffer, solutions and samples were pre-filtered with a 0.22 µm filter. As derivatizing agent, OPA (o-Phthaldialdehyde reagent, Merck) was chosen for its capacity to bind amino groups and act as fluorophore. The injection volume was 10 µL. The signal used to visualize the fluorescence was set at 338 nm bandwidth 10 nm with a reference wavelength of 390 nm bandwidth 20 nm. Spectra were collected during analysis in a range between 200 nm and 500 nm with a step of 2 nm, to have a side control and make identifications easier. All data were displayed and analysed on Agilent ChemStation software.

### 2.5. Plant Morphometry, Photosynthetic Efficiency, and Phytochemistry

Data deriving from the field activity were analyzed through the software R(Version 3.3.3 © 2019–2016) and particularly by the lme4 and glmmTMB, mgcv and gamm4 package. Linear Mixed Effect Models (LME) or Generalized Linear Mixed Effect Models (GLMM) were initially applied.

However, when considering trends in time of morphological parameters, model validation confirmed non-linear pattern in the residuals, therefore, the application of GAMMs (Generalized Additive Mixed Models) was required after confirming through the AIC evaluation.

Specifically, canopy was assumed to be Gamma distributed (data were considered Gamma-distributed because of the occurrence of negative fitted values), number of leaves and flowers was considered Poisson distributed but they were then switched to a negative binomial distribution to deal with overdispersion. Concerning the evaluation of F_v_/F_m_ ratio from PEA measurements, only data from the 5th to 7th survey were provided, so the (b) model was directly run. This ratio is an index, therefore it was assumed to be beta-distributed. Models were run providing for a double random effect, which is the individual nested within the plot. Model validation was performed by plotting residuals from each model against fitted values as well as each covariate and random component. Because of the high tendency of violation of independence as a consequence of temporal correlations (data were collected week by week), a corARMA1 correction was provided for each model [22]. The selection of the best model was provided by following the Aikaike Criterion (AIC) through the anova function.

Concerning plant chemical parameters, TPC, TSC, and total amino acidic content were analyzed through a GLMM as above, considering the plot as random factor (R, Version 3.3.3 © 2019–2016 and particularly by the lme4 and glmmTMB package). Data were assumed to be binomially distributed but necessitated a switch to a beta-binomial distribution due to over-dispersion. The fixed effect was time in interaction with the management condition (tillage, irrigation) in order to evaluate their effect on response variables in time by exploiting 95% confidence bands.

### 2.6. Soil Properties

The soil C and N stock (Mg ha^−1^) at 0–30 cm depth was calculated as follows: profile soil stock (Mg ha^−1^) = (soil C/100) × BD (Mg m^−3^) × depth (m) × 10,000 (m^2^ ha^−1^). Likewise, soil N stock on the soil was determined.

Data on soil C and N (both concentration and stock), as well as on grain yield and straw biomass of cowpea, were subjected to analysis of variance (ANOVA) with a split plot design following procedures outlined by Gomez and Gomez [23] and using the “agricolae” package of RStudio 3.3.3. The main-plot factor was the tillage system (NT vs. CT), while the subplot factor was water management (W vs. NW plots). When the Shapiro–Wilk test and the Levene’s test did not confirm the assumptions of ANOVA, data were log-transformed before analysis. Tukey’s honestly significant difference (HSD) as a post hoc was used to test for significant differences in variables among treatments with a *p*-value of 0.05 as the threshold for statistical significance.

## 3. Results

### 3.1. Biomass and Grain Yield

**Figure 2:**
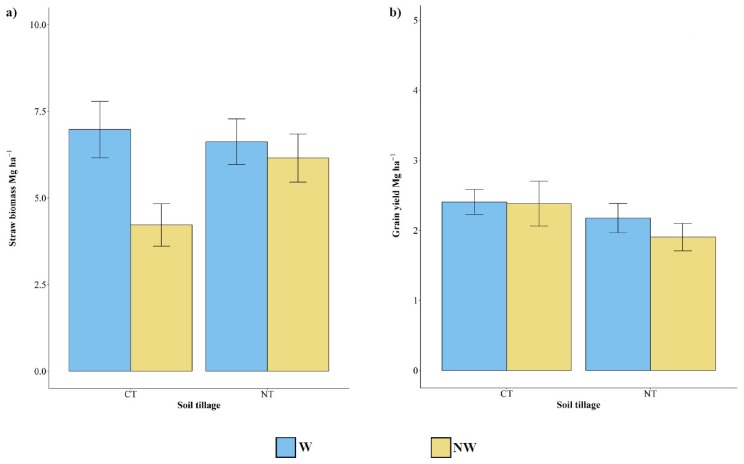
(**a**) Cowpea biomass and (**b**) grain yields (Mg ha^−1^). Values are mean ± SEM. CT = conventional tillage, NT = no tillage, W = watered, NW = not watered.

No significant differences were detected between grain yield on plant grown under NT and CT conditions (Figure 2) and with and without irrigation. Concerning the straw biomass, significant higher production in the W plots than in the NW ones (6.78 vs. 5.18 Mg ha^−1^; +31%) was observed. Conversely, no difference was found between CT and NT plots (Figure 2).

The interaction between the tillage system and water supply showed significant differences in cowpea straw biomass while not in the grain yield production (Figure 2). In detail, CT-W and NT-W had the highest straw biomass with 6.94 and 6.63 Mg ha^−1^, respectively. CT-NW had the lowest straw biomass (4.22 Mg ha^−1^), while NT-NW did not show a significant difference compared to the other conditions (Figure 2).

### 3.2. Morphometrics and Growth Parameters

Planting density was measured in all plots and subplots at the late flowering stage (R4-R5) and was as follows: under CT, the W (CT-W) and the NW (CT-W) subplots had an average of 16.8 and 15.3 plants m^−2^, respectively; under NT, the W (NT-W) and the NW (NT-NW) subplots had on average value of 18.5 and 17.5 plants m^−2^, respectively.

**Figure 3:**
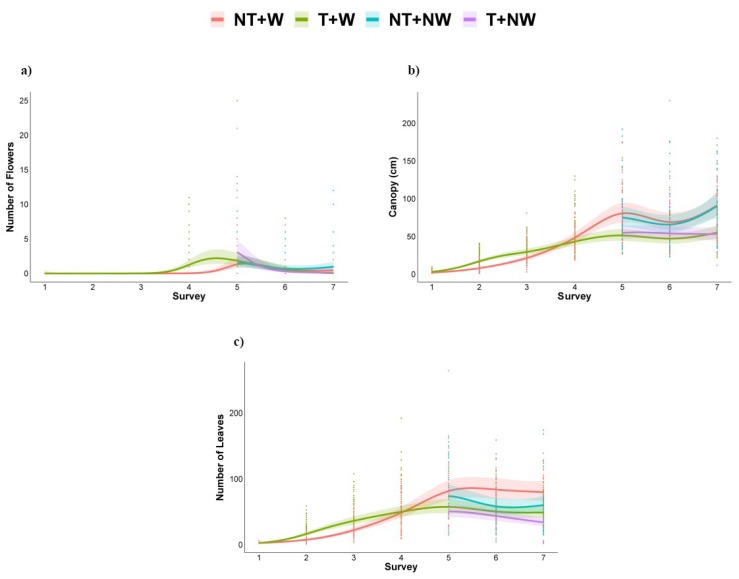
Models showing the trend of (**a**) number of flowers, (**b**) canopy length, and (**c**) number of leaves during the experimental period in the four treatments. CT=conventional tillage, NT=no tillage, W = watered, NW = not watered.

Figure 3 shows the results related to the morphometric parameters detected: number of flowers (intended as a reproductive parameter), dimension of the canopy, and total number of leaves (intended as growth parameters). The flowering period took place starting from the 4th week. As Figure 3a shows, CT plots revealed a sudden blooming followed by a likewise sudden interruption, while NT plots showed a more contained but constant blooming. Therefore, blooming was significantly higher between the 4th and the 5th week in CT plots and then significantly higher in NT plots. No effects were due to no irrigation (see 95% confidence bands). Concerning vegetative parameters, the obtained data (Figure 3b,c) suggested that a significant difference resides in the decrease of the total number of leaves caused by the absence of irrigation at the 6th week, followed by recovery during the last survey. As far as tillage is concerned, it was found that at the beginning of the growth season (from the 1st to the 3rd week) both plants canopy expansion and total number of leaves were significantly higher in CT, then during the 4th survey, no differences were detected between the two groups, while from the 5th week to the end NT plots showed a significantly higher performance for both the considered parameters.

### 3.3. Efficiency of Photosynthesis and Metabolic Profile

**Figure 4:**
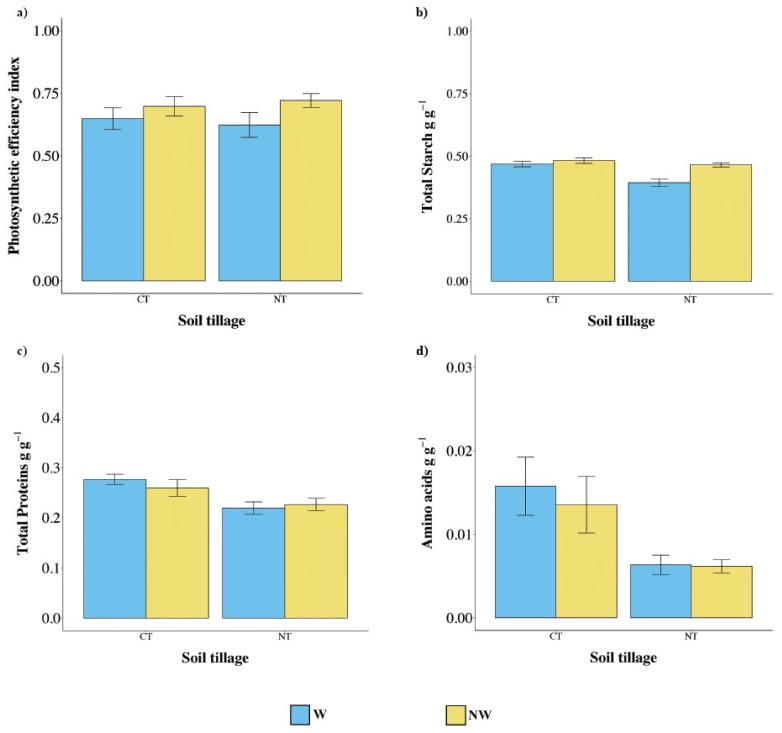
(**a**) Leafy photosynthetic efficiency, (**b**) TSC, (**c**) TPC, and (**d**) amino acids. Values are mean ± SEM. CT = conventional tillage, NT = no tillage, W = watered, NW = not watered.

Figure 4 shows the results relative to photosynthetic efficiency, TSC, TPC, and amino acid content. Results suggested that metabolic features were lowly affected by tillage management. Photosynthetic efficiency (Figure 4) was comparable between the two treatments (χ^2^ = 5.03, *p* = 0.17). Concerning proteins (Figure 4b), TPC was not significantly influenced by treatments (χ^2^ = 6.14, *p* = 0.11).

Also, the total amino acidic content (Figure 4d) did not show any significant difference between treatments (χ^2^ = 4.15, *p* = 0.25), with an average amount ranging between 0.5% and 2% of the dry matrix.

Finally, TSC was clearly influenced by treatments (χ^2^ = 29.63, *p* < 0.001). In particular, both CT (β = 0.3 ± 0.1, *p* = 0.004;) and NW (β = 0.29 ± 0.05, *p* < 0.001) caused an increase in the TSC of about 4.5% and 3.3%, respectively. The interaction resulted to be significant as NT coupled to W caused the most dramatic decrease in TSC (β = −0.23 ± 0.06, *p* < 0.001).

### 3.4. Soil Organic Carbon and Total Nitrogen

Concerning soil chemical characteristics, a significant difference in C and N concentration occurred between NT and CT, especially at the topmost (0–5 cm) soil horizon. In detail, C and N concentrations in this soil layer were 59% and 27% higher under NT than under CT. No significant difference in soil C and N concentration was found in the 5–30 cm soil layer. (Table 1 and Table 2). Overall, soil C stock in the 0–30 cm soil layer was statistically affected by the tillage system and was 2.58 Mg ha^−1^ higher in NT than in CT soil (+5%). Conversely, soil N stock in the same soil layer did not statistically differ between NT and CT, even though NT tended to increase soil N stock value by 4% (Table 1 and Table 2).

## 4. Discussion

### 4.1. Suitability of V. unguiculata for Mediterranean CA

Our findings, overall, suggest that tilling is not fundamental to guarantee cowpea growth and yield. Therefore, cowpea is a crop suitable for CA practices and could be cultivated in harsh conditions such as arid and semiarid regions, where not all the crops perform well. For instance, grain yield in common bean *Phaseolus vulgaris* L. is highly affected (−70%) under drought conditions [24].

Usually, tillage alters the physical-chemical properties of soil, and NT vs. CT may greatly impact plant growth and yield [25]. Unfavorable effects of NT on crop yield have been widely reported immediately after the conversion from CT [26]. This is because NT may increase soil strength and BD in the initial years due to transient compaction of soil [27], thus reducing the root growth of plants [28]. However, it has also been shown that negative effect usually expires from three to five years after the conversion from CT to NT [19]. Our experimental activities with seven-year NT corroborated this improvement of yield potential under NT in the medium-long term.

No-till is usually indicated to enhance the water content of soil [29], which is of primary importance to sustain crop yield, especially under arid and semi-arid climates [30]. Our data show that NT tended to mitigate soil water losses compared with CT and cowpea tolerated NW conditions well under NT.

These results are in line with earlier studies reporting the high potential of NT to enhance crop yield in non-irrigated field management [31]. The efficacy of cowpea to grow under water deficient conditions and with reduced or no tillage was also documented by Moroke et al. [32] in the experimental area of Texas, and by Ahamefule and Peter [33] in Nigeria. Moroke and co-workers [32] suggested that the cultivation of this species enhances the residual soil water and the presence of surface residue management due to cover crops and NT systems usually increases the whole stored soil water.

In the Mediterranean basin, mean temperatures are constantly increasing and precipitation pattern is changing towards hot and dry summer seasons as a consequence of climate change. In this context, European production of pulses has been constantly declining (from 5.8 to 1.8 million ha from 1961 to 2013), mainly due to the introduction of more specialized and intensive crops such as wheat, rice, and corn [34,35,36], but also as a consequence of productive instability of highly water-demanding species [37]. However, the European Commission [38,39] welcomes initiatives to increase the EU’s plant protein production in a sustainable and agro-ecological way. Since cowpea can be considered as a leguminous species with reduced water demand and high drought tolerance [40], it may be considered to support the cultivation of legumes in Europe replacing currently cultivated species that have higher water demands [37]. In addition, we indicate that cowpea is also a reliable alternative to the common Mediterranean bean (*P. vulgaris*) in North-African countries, especially because of high drought tolerance during the reproductive phase [41]. Therefore, combining NT through the cultivation of species and cultivars of pulses highly resistant to water stress (such as the cowpea) could support the resumption of legume cultivation in Europe and in the Mediterranean basin to deal with the ongoing claims about pulses and climate change [42].

Concerning the growth parameters, our experiment shows that plants initially grow better on CT soils, but after having joined a critical dimension the NT treatment helps to maintain and stimulate a more pronounced growth in a significant manner. This is particularly true if soil treatment is associated with NW. Generally, the two treatments on the global pattern are not significantly different, but this is due to a balance on the whole values associated with pretty large variations during the growing season. Also, leafy photosynthetic efficiency did not differ between treatments. Therefore, we highlight a great capability of the cowpea to grow under reduced tillage and low water regime.

Many studies focused on the impact of CA on crop yields [6], while no attention was given to morphological adaptations and metabolic profiling. The latter is an important aspect, impacting on nutritional importance and sustainability of this crop.

Generally, the main constituents of the seeds were not strongly affected by treatments (NT and NW) with the exception of TSC. TSC is normally related to a plant’s ability to photosynthesize, therefore, some studies conducted on *V. unguiculata* seeds showed a reduction in TSC under NW, also in order to increase the number of free analytes able to gather water through osmosis [43]. Here, we found an increase in TSC in NW conditions. Maybe this could be related to the maintenance of a high photosynthesis rate, also without irrigation. As a matter of fact, NW is not always related to starch degradation in plant tissues [44]. These data further confirm a good resistance of this species against drought, a condition highly dangerous for many crop species not equally able to adapt to climate changes [45]. Finally, in terms of protein and amino acids, our analyses confirm that cowpea is an important source of these nutritional components and the growing condition did not affect their amount. Considering that in the Mediterranean area, especially in the Eastern and African sides, diets are deficient in terms of protein and amino acid intake [16,46] and despite the change in food regimes that lead to a decrease of the intake of animal proteins, we estimate that cowpea could be a great support or even a crucial aliment to compensate these lacks. Moreover, the cowpea amino acidic content is two times higher than the widespread common bean *P. vulgaris* [16], therefore this species could be a good substitute for the traditional legume crops.

### 4.2. Conservative Cultivation of V. unguiculata Enhances Soil Fertility

As well documented, Fabaceae are able to accumulate organic nitrogen thanks to symbiotic interactions at the rhizosphere level. This phenomenon has positive effects also on soil fertility; however, soil management could also affect organic and inorganic components. For instance, CT is considered a major cause of soil C and N depletion as a consequence of soil organic matter mineralization [47]. NT has been widely indicated as a key strategy to increase C storage in arable soils. Our results are in line with this consideration as they show that converting CT to NT increased C stock by 2.58 Mg ha^−1^ on a silty clayey soil (Table 1), which means that NT increased the potential of soil to sequester C by 0.32 Mg ha^−1^ yr^−1^ under our experimental conditions. This is consistent with the previous findings of a review study by West and Post [48]. These authors reported that NT may increase C sequestration in soil by 0.20–0.57 Mg ha^−1^ yr^−1^, according to the complexity of crop rotation. Also Aguilera et al. [49], in a recent meta-analysis reported a 0.44 Mg ha^−1^ yr^−1^ higher soil C sequestration under NT in the surface 34 cm of soil under a temperate climate.

Our findings also suggested that considering soil C expressed as concentration (g kg^−1^) instead of as mass (Mg ha^−1^) may lead to overestimating the role of NT for soil C sequestration. However, we underline that we have data from the last eight years about the used NT soil patches, and our surveys showed an increase in soil C concentration in the 0–30 cm soil layer of, on average, 11% compared with CT. Conversely, soil C stock, which takes into account soil BD, was only 5% higher under NT than under CT. This is because lower BD of soil in the surface layers under NT (especially 0–5 cm) reduces the actual impact on soil C accumulation [18]. Nevertheless, NT did not reduce soil C stock in the subsurface soil layers (5–30 cm), and the net impact of NT on soil C stock in the 0–30 cm soil was positive.

Concerning nitrogen, the soil tillage could increase soil oxygen exposure and this promotes soil organic matter mineralization and soil N depletion [50]. Therefore, increasing soil organic matter in soils is a key way not only to increase soil C stock and mitigate climate change but also to enhance soil fertility and thus sustain food production [51].

As expected, our results showed that the evolution in soil N concentration and stock followed a similar pattern to that of soil C levels. Converting CT to NT led to a significant increase of soil N concentration in the 0–5 cm soil layer (Table 2). These results confirmed that variation of soil C and N levels as induced by NT differed considerably depending on the surface (0–5 cm) or subsurface (5–30 cm) soil layers [52]. Mazzoncini et al. [53] also found that soil C and N accumulated under NT may be mainly attributed to soil C and N variation in the topmost (0–10 cm) soil layer, which is in substantial agreement with our results (Table 1 and Table 2). This is mainly due to the fact that NT limits the direct input of fresh organic matter to the subsoil, thus reducing the downward movement of soil organic matter, which is usually increased only in the surface 10 cm of soil. In addition, reducing soil disturbance decreases N mineralization and losses especially in the topmost soil layers [53], due to a lower temperature [54] and aggregate turnover [55] in non-tilled soils than in tilled ones.

## 5. Conclusions

In conclusion, CA can offer many advantages in the Mediterranean context. First of these, is the saving of energy and costs. Studies performed on cowpea in a semiarid area of India showed that zero tillage practices provide a considerable energy saving (−17.1 GJ ha^−1^) due to the lower input compared to CA [56]. The energy efficiency should be about 13 times higher in zero tillage systems than in conventional ones. However, Dixit et al. [56] showed that cowpea yield (intercropped with sorghum) was significantly higher in the context of conventional agriculture compared with NT.

Another key point of CA is the ability to reduce the impact on the greenhouse effect by anthropogenic gases emission. As suggested by Powlson [18] the total carbon sequestration in NT condition could reach about 0.3 Mg ha^−1^ yr^−1^; our data confirm that the total CO_2_ sequestration per year was estimated to be 0.32 Mg yr^−1^.

This is due also to the usage of a cover crop mixture during the winter period which provided for better capture of the CO_2_, while in Powlson [18] the meta-analysis took into account predominantly field not managed with cover crops. This underlines the importance of the integration of cover crops in zero tillage management.

Finally, concerning the ability of the soil to save water, our data suggested an increase of SOC in NT area against CT. Previous studies suggested that in the context of the Mediterranean geographical area, an increase of about 0.4% SOC may lead to an increase of up to 34% of water saving [57].

Regarding all these elements and knowing that cowpea is a very interesting stress-tolerant minor crop with a short time maturity (about 60 days), cowpea could be introduced not only to relieve and reduce agricultural impact and climatic changes but also to supply a lot of vegetable-derived nutrients, like proteins and amino acids.

## Figures and Tables

**Table 1:** Soil organic carbon. Values are mean ± SEM. Significance levels: * < 0.05, ** < 0.01, *** < 0.001.

Condition	Code	C Concentration 0–5 cm (g C kg^−1^ soil)	C Concentration 5–30 cm (g C kg^−1^ soil)	C Stock (Mg ha^−1^)
Tillage	CT	12.49 ± 1.48	12.39 ± 0.88	48.56 ± 3.37
	NT	19.92 ± 0.73	12.5 ± 0.76	51.15 ± 2.24
Signif.		***	n.s.	*
Water	W	16.17 ± 4.06	12.49 ± 0.76	49.98 ± 2.81
	NW	16.24 ± 4.22	12.4 ± 0.88	49.73 ± 3.51
Signif.		n.s.	n.s.	n.s.
Interaction	CT-W	12.46 ± 0.92	12.43 ± 1.03	48.69 ± 3.53
	CT-NW	12.51 ± 2.07	12.34 ± 0.87	48.43 ± 3.74
	NT-NW	19.88 ± 0.97	12.54 ± 0.54	51.27 ± 1.24
	NT-NW	19.96 ± 0.55	12.45 ± 1.02	51.03 ± 3.19
Signif.		n.s.	n.s.	n.s.

**Table 2:** Nitrogen content in the soil. Values are mean ± SEM. Significance levels: * < 0.05, ** < 0.01, *** < 0.001.

Condition	Code	N Concentration 0–5 cm (g N kg^−1^ soil)	N Concentration 5–30 cm (g N kg^−1^ soil)	N Stock (Mg ha^−1^)
Tillage	CT	1.49 ± 0.21	1.49 ± 0.1	5.84 ± 0.46
	NT	1.9 ± 0.21	1.57 ± 0.1	6.1 ± 0.37
Signif.		**	n.s.	n.s.
Water	W	1.72 ± 0.37	1.55 ± 0.11	6.04 ± 0.39
	NW	1.67 ± 0.22	1.52 ± 0.12	5.91 ± 0.48
Signif.		n.s.	n.s.	n.s.
Interaction	CT-W	1.41 ± 0.09	1.48 ± 0.1	5.76 ± 0.37
	CT-NW	1.57 ± 0.28	1.5 ± 0.12	5.93 ± 0.58
	NT-NW	2.03 ± 0.22	1.62 ± 0.06	6.32 ± 0.08
	NT-NW	1.76 ± 0.08	1.53 ± 0.13	5.88 ± 0.43
Signif.		n.s.	n.s.	n.s.

## Supplementary Materials

The following are available online at https://www.mdpi.com/2223-7747/9/1/48/s1, Figure S1: Evolution of daily precipitation (bars) and average daily temperature (line) of the field site during the course of the experiment.

## Abbreviations

CA: conservation agriculture; CT: conventional tillage; NT: no-tillage; W: watered; NW: not watered.
