# Supplementary material for: Sustainability Perspectives of Vigna unguiculata L. Walp. Cultivation under No Tillage and Water Stress Conditions
Source: Plants (Basel). 2019 Dec 30;9(1):48. doi: 10.3390/plants9010048 (PMC7020161; doi:10.3390/plants9010048)
Supplement: Supplementary file 1 [file plants-09-00048-s001.pdf]

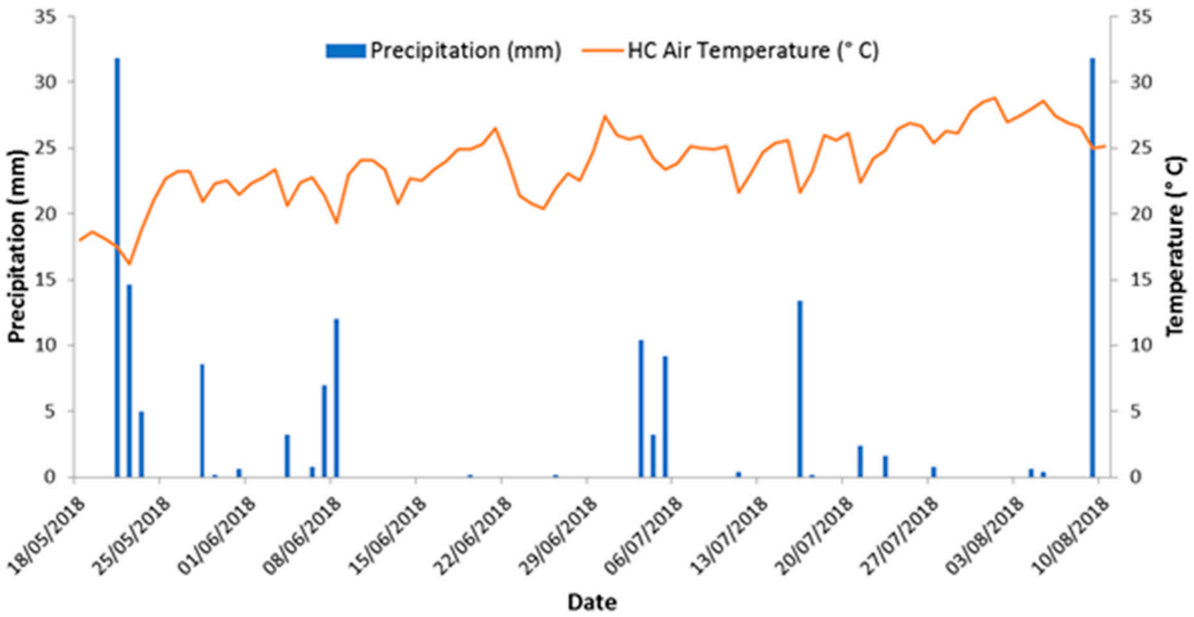

Figure S1: Evolution of daily precipitation (bars) and average daily temperature (line) of the field site during the course of the experiment.
